# A GATA Transcription Factor from Soybean (*Glycine max*) Regulates Chlorophyll Biosynthesis and Suppresses Growth in the Transgenic *Arabidopsis thaliana*

**DOI:** 10.3390/plants9081036

**Published:** 2020-08-15

**Authors:** Chanjuan Zhang, Yi Huang, Zhiyuan Xiao, Hongli Yang, Qingnan Hao, Songli Yuan, Haifeng Chen, Limiao Chen, Shuilian Chen, Xinan Zhou, Wenjun Huang

**Affiliations:** 1 Key Laboratory of Biology and Genetic Improvement of Oil Crops, Ministry of Agriculture and Rural Affairs, Oil Crops Research Institute of Chinese Academy of Agricultural Sciences, Wuhan 430062, China; zhangchanjuan@caas.cn (C.Z.); huangyi@oilcrops.cn (Y.H.); xiaozhiyuan@caas.cn (Z.X.); yanghongli@caas.cn (H.Y.); haoqingnan@caas.cn (Q.H.); songliyuan@caas.cn (S.Y.); chenhaifeng@caas.cn (H.C.); chenlimiao@caas.cn (L.C.); chenshuilian@caas.cn (S.C.); 2 Key Laboratory of Plant Germplasm Enhancement and Specialty Agriculture, Wuhan Botanical Garden, Chinese Academy of Sciences, Wuhan 430074, China; 3 Innovative Academy of Seed Design, Chinese Academy of Sciences, Beijing 100101, China

**Keywords:** *Glycine max*, soybean, GATA, transcription factor, chlorophyll, photosynthesis, growth, nitrogen

## Abstract

Chlorophyll plays an essential role in photosynthetic light harvesting and energy transduction in green tissues of higher plants and is closely related to photosynthesis and crop yield. Identification of transcription factors (TFs) involved in regulating chlorophyll biosynthesis is still limited in soybean (*Glycine max*), and the previously identified *GmGATA58* is suggested to potentially modulate chlorophyll and nitrogen metabolisms, but its complete function is still unknown. In this study, subcellular localization assay showed that GmGATA58 was localized in the nucleus. Histochemical GUS assay and qPCR assay indicated that *GmGATA58* was mainly expressed in leaves and responded to nitrogen, light and phytohormone treatments. Overexpression of *GmGATA58* in the *Arabidopsis thaliana* ortholog *AtGATA21* (*gnc*) mutant complemented the greening defect, while overexpression in Arabidopsis wild-type led to increasing chlorophyll content in leaves through up-regulating the expression levels of the large of chlorophyll biosynthetic pathway genes, but suppressing plant growth and yield, although the net photosynthetic rate was slightly improved. Dual-luciferase reporter assay also supported that GmGATA58 activated the transcription activities of three promoters of key chlorophyll biosynthetic genes of soybean in transformed protoplast of Arabidopsis. It is concluded that *GmGATA58* played an important role in regulating chlorophyll biosynthesis, but suppressed plant growth and yield in transgenic Arabidopsis.

## 1. Introduction

Photosynthesis, a major contributor to crop yield, predominantly takes place in leaves where chlorophyll, one of the most abundant biological molecules in higher plants, plays unique and essential roles in photosynthetic light harvesting and energy transduction [1]. It has been reported that chlorophyll concentration, specific leaf weight or area and photosynthetic rate have impacts on crop yield, including soybean [2,3,4]. Among them, photosynthetic rate in leaves highly correlates with crop yield [5]. Thus, it is suggested that improving photosynthetic efficiency in soybean through biotechnology is a promising alternative way to increase crop yield [6]. Further, it was previously documented that the photosynthetic rate of soybean was highly correlated with chlorophyll content in leaves [7]. An increase in chlorophyll content provides an improved capacity to convert light energy to chemical energy and enhanced carbohydrate accumulation [8]. Therefore, it is possible to improve photosynthesis rate and crop yield through increasing chlorophyll content in leaves.

Chlorophyll consists of a chlorin ring and a geranylgeranyl diphosphate-derived isoprenoid, which are produced by tetrapyrrole and methylerythritol phosphate (MEP) biosynthetic pathways, respectively [9]. To date, the chlorophyll biosynthetic pathway has been clearly elucidated and almost all genes encoding enzymes involved in this pathway have been identified in Arabidopsis [1]. Moreover, multiple environmental stimulants and endogenous effectors are found to control the expressions of chlorophyll biosynthetic genes, but the transcription factors (TFs) involved in the regulation of chlorophyll biosynthetic pathway are little reported [10,11]. There are only several TFs found to date, primarily from Arabidopsis to regulate chlorophyll biosynthesis, including LONG HYPOCOTYL 5 (HY5), PHYTOCHROME-INTERACTING FACTORs (PIFs), GOLDEN2-LIKE (GLK) and GATA TFs [10,11]. Most of them are also involved in the light signaling pathway and photomorphogenesis, such as HY5 and PIFs that regulate chlorophyll biosynthesis through binding to G-box-containing promoter regions of certain key chlorophyll pathway genes [11]. Two renowned members of class B GATA TFs (B-GATAs), GNC (GATA, NITRATE-INDUCIBLE, CARBON METABOLISM-INVOLVED) and its paralogous gene GNL/CGA1 (GNC-LIKE/CYTOKININ-RESPONSIVE GATA TRANSCRIPTION FACTOR1; referred to hereafter as GNL) have been identified in Arabidopsis to regulate chlorophyll biosynthesis and chloroplast formation [12,13,14].

GATA TFs are evolutionarily conserved proteins that contain a characteristic type IV zinc-finger DNA-binding domain and recognize a consensus sequence (A/T)GATA(A/G) of target promoter regions [15]. In plants, they play diverse roles in developmental control, responses to environmental stresses, hormones as well as nitrogen metabolism [16]. For example, a poplar *GATA* gene, *PdGATA19*/*PdGNC* was recently identified to not only regulate chlorophyll biosynthesis and also promote photosynthetic rate and plant growth [17]. Although the GATA gene family has been identified in Arabidopsis [15,18], rice [15], grape [19] and soybean [20], but only members have been functionally characterized to date. In soybean, the *Glyma12g08131* encoding a GATA factor has been found to be possibly involved in nitrogen metabolism [21]. Nitrogen is essential to crop growth and yield, and nitrogen metabolism is closely related to chlorophyll metabolism and photosynthesis [22,23]. Moreover, nitrogen status in the leaves of several major crops, including soybean, can be rapidly, precisely and easily determined by a chlorophyll meter which reflects chlorophyll content [24,25]. Recently in our lab, two GATA members, *GmGATA44* and *GmGATA58* from a genome-wide analysis of GATA TFs family in soybean were found to be potentially involved in regulation of nitrogen and chlorophyll metabolisms [20]. *GmGATA44* had been demonstrated to genetically complement the reduced chlorophyll phenotype of the Arabidopsis *gnc* mutant. It is a pair of paralogous genes *GNC* and *GNL* that contribute to chlorophyll biosynthesis and chloroplast formation in Arabidopsis seedlings [14]. However, the biological role of *GmGATA58*, a paralogous gene with *GmGATA44*, in chlorophyll biosynthesis, photosynthetic rate, growth and yield is still unknown. Therefore, in this study, the function of *GmGATA58* TF was fully characterized to provide new insights into understanding the regulatory mechanism of chlorophyll biosynthesis and a potential tool gene for improving soybean growth and yield through biotechnology in future.

## 2. Results

### 2.1. Sequence Analysis of GmGATA58

The full-length cDNA of *GmGATA58* gene was amplified from soybean variety ‘TianLong No.1′ and it contained an entire ORF (open reading frame) of 969 bp, encoding a protein of 322 amino acid residues with a predicted molecular weight of 36.2 kDa and pI of 9.49. GmGATA58 contained the highly conserved GATA zinc finger domain and also a conserved leucine-leucine-methionine (LLM) domain closely located at the C-terminus as GmGATA44, AtGNC and AtGNL factors [26] (Figure S1). *GmGATA58*, identified previously as a member of B-GATA TF family [20], was further classified into the long B-GATA subfamily according to the structural criteria described by Behringer et al. [26] due to the long region of the N−terminal to the GATA domain. In addition, GmGATA58 showed a high similarity with GmGATA44 (88% identity) at amino acid level, but only 44% and 41% identities with AtGNC and AtGNL protein, respectively. However, within the conserved GATA domain region, GmGATA58 shared 76% and 70% identities with AtGNC and AtGNL, respectively.

### 2.2. GmGATA58 Localizes in Nucleus

**Figure 1:**
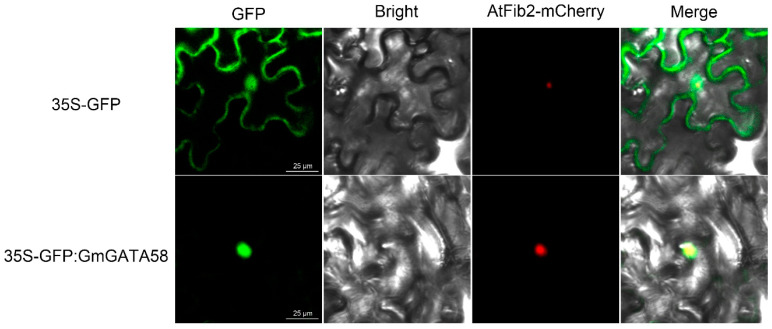
Subcellular localization of GmGATA58 protein in leaves of *Nicotiana benthamiana* using *Agrobacterium*-mediated transient expression. The plasmid construct containing GFP alone or GFP::GmGATA58 fusion protein was co-transformed into leaves of *N. benthamiana* with AtFib2-mCherry construct (nucleolar marker) and fluorescence signals were visualized using a confocal laser scanning microscopy 3 d after infiltration. GFP—(green), mCherry—(red), white—and their merge images were shown. Scale bar = 25 μm.

In eukaryotes, TFs are produced in cytoplasm but function in nucleus to regulate the transcription of downstream genes. TFs are generally transported from cytoplasm to nucleus via nuclear localization signal (NSL). Correspondingly, GmGATA58 as a TF was predicted bioinformatically to contain a putative NLS and thus localize in nucleus (Figure S1). In order to further confirm this prediction, the subcellular localization of GmGATA58 in leaves of *Nicotiana benthamiana* was analyzed using *Agrobacterium*-mediated transient expression. The results showed the GFP::GmGATA58 fusion protein driven by the CaMV 35S promoter produced a strong green fluorescent signal only in the nucleus, which completely overlaid the red fluorescence produced from AtFib2-mCherry, a well-known nucleolar marker [27] (Figure 1). Meantime, the positive control GFP protein alone driven by the CaMV 35S promoter produced strong green fluorescent signals in whole cells (Figure 1). These results suggested that GmGATA58 was indeed localized in nucleus.

### 2.3. Expression Pattern of GmGATA58

**Figure 2:**
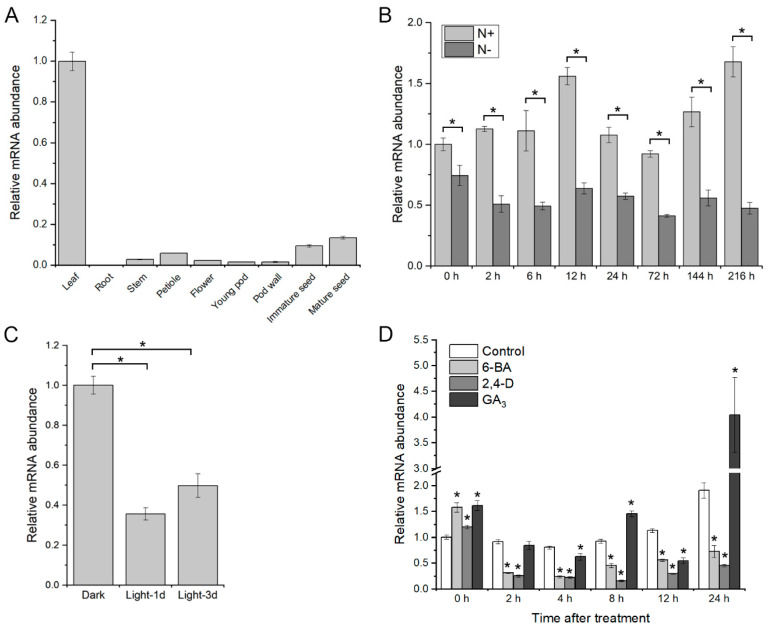
Expression pattern of *GmGATA58* in various tissues of soybean (**A**) and in response to nitrogen (**B**), light/dark (**C**) and three phytohormones treatments (**D**). A, it is noted that pod wall sample are collected from immature pod. B, soybean seedlings were grown in the 7.5 mM solution of normal nitrogen (N+) or the 0.75 mM solution of low nitrogen (N−) conditions for up to 9 days. C, soybean seedlings were grown in continuous dark condition for 3 days and then transferred to continuous light condition for up to 3 days. D, the 15-day-old seedlings were sprayed with 100 μM 6-BA, 100 μM 2, 4-D or 100 μM GA_3_ phytohormones and distilled water as the control. The leaves of seedlings treated with nitrogen, light and phytohormones were harvested for qPCR assay at the different times. The *GmACT11* gene was used as a reference gene and column values represented mean ± SD (standard deviation). Asterisks (*) represent significant difference between groups or against the control in hormones treatment at the level of *p* < 0.05.

Expression pattern of *GmGATA58* in various tissues of soybean was firstly investigated by qPCR assay. The result indicated that *GmGATA58* was predominately expressed in leaves, relatively moderately in immature and mature seed and very lowly in other tissues (Figure 2A). *GmGATA58* and *GmGATA44* were previously found to respond to nitrogen stress [20]. Here, *GmGATA58* expression under the 7.5 mM solution of normal nitrogen (N+) and the 0.75 mM solution of low nitrogen (N−) conditions was also analyzed (Figure 2B). Although the expression level of *GmGATA58* did not change considerably throughout the whole stages of either N+ or N− treatment, but it was significantly higher under N+ condition than that under N− condition at each sampling point, and their greatest difference between N+ and N− treatments occurred at 216 h after treatment (Figure 2B). In other words, *GmGATA58* was largely expressed under normal nitrogen condition and suppressed by low nitrogen stress. In addition, *GmGATA58* expression under light and phytohormones treatments was also investigated as a number of *cis*-acting regulatory elements involved in light and phytohormones responses were predicted in its promoter sequence (Table S1). The results showed that *GmGATA58* mRNA abundance considerably declined in leaves of soybean seedlings when seedlings removed out of a 3-day dark treatment were exposed to light treatment for 1 day and 3 days (Figure 2C). In response to 6-BA and 2, 4-D treatment, *GmGATA58* transcript level decreased largely from 2 to 24 h after treatments (Figure 2D), but under GA_3_ treatment *GmGATA58* expression level increased at 8 and 24 h, particularly at 24 h with the maximum up-regulation (Figure 2D), compared to the water control. In short, *GmGATA58* was highly expressed in leaves and responded to nitrogen level, light and phytohormone treatments.

### 2.4. Histochemical GUS Assay of Transgenic Arabidopsis Expressing GmGATA58 Promoter

**Figure 3:**
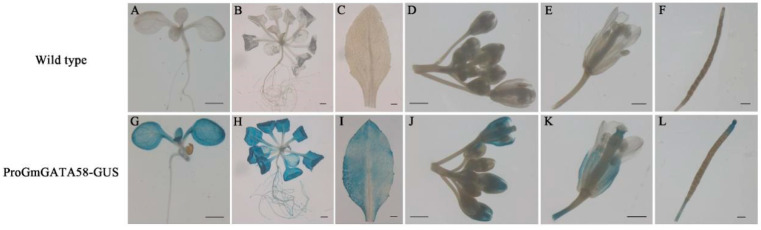
Histochemical GUS assay of transgenic Arabidopsis plants. The wild-type plant (**A**–**F**) and transgenic plant (**G**–**L**) carrying *GmGATA58* promoter-GUS construct are indicated for GUS staining. Scale bars = 0.1 cm.

The preferable expression of *GmGATA58* in soybean leaves was demonstrated by the qPCR results above. The histochemical GUS assay was also carried out to further analyze the spatial and temporal expression pattern of *GmGATA58* in transgenic Arabidopsis expressing the *GmGATA58* promoter. The results indicated that no GUS activities were detected in any tissue of wild-type plant as expected (Figure 3A–F), while in transgenic plants carrying the *GmGATA58* promoter showed a different spatial and temporal expression pattern (Figure 3G–L). *GmGATA58* was strongly expressed in cotyledons and young true leaves of both the 5- and 13-day-old seedlings, but weakly expressed in their root and rarely expressed in hypocotyls (Figure 3G,H). In transformed adult Arabidopsis plants, the expression of *GmGATA58* was strongly detected in rosette leaves (Figure 3I), but lowly expressed in reproductive tissues, such as silique and flower except sepal with a moderate expression (Figure 3J–L). Overall, *GmGATA58* was strongly expressed in leaves throughout all developmental stages of plants.

### 2.5. GmGATA58 Genetically Complements the gnc Mutant of Arabidopsis

**Figure 4:**
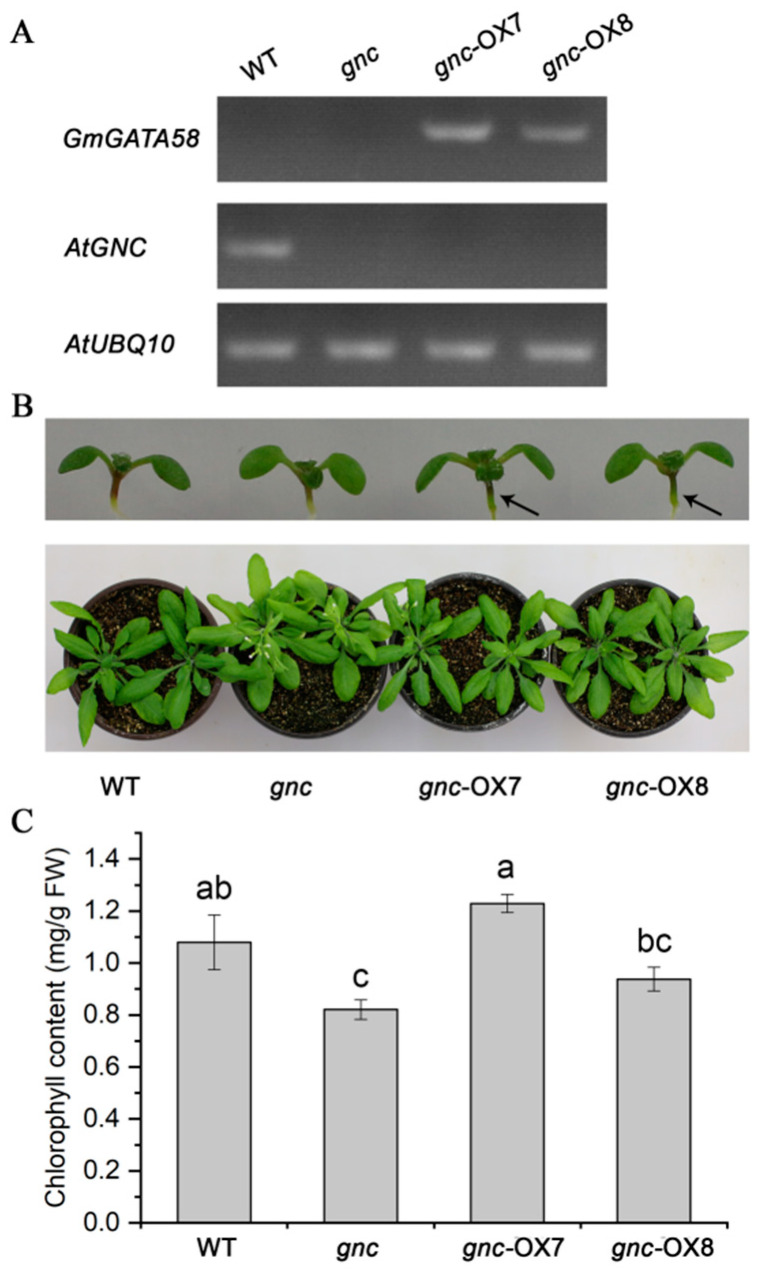
Genetical complementation of *GmGATA58* gene in the Arabidopsis *gnc* mutant. (**A**) Semi-quantitative RT-PCR assay of *GmGATA58* and *AtGNC* gene in the 3-week-old rosette leaves of wild-type (WT), *gnc* mutant, and two T_3_ transgenic lines overexpressing *GmGATA58* in the *gnc* mutant background (*gnc*-OX7 and *gnc*-OX8). The *AtUBQ10* gene was used as an internal control. (**B**) Phenotypic comparison of wild-type, *gnc* mutant, and two transgenic lines at one week (upper panel) and four weeks (bottom panel) after germination. Arrows indicated dark green hypocotyls. (**C**) The chlorophyll content measurement in the rosette leaves of wild-type, *gnc* mutant and two transgenic lines at three weeks after germination. Data are presented as mean ± SD (*n* = 10) from three independent measurements. Means not sharing any lowercase letter are significantly different by the Tukey test at the 5% level of significance.

It was previously suggested that both *GmGATA44* and *GmGATA58* were likely to be involved in regulation of nitrogen and chlorophyll metabolisms in soybean, and the former had been demonstrated to regulate chlorophyll biosynthesis by genetic complementation of the Arabidopsis *gnc* mutant [20]. Therefore, overexpression of *GmGATA58* under the control of CaMV 35S promoter in the *gnc* mutant background was also performed. Two independent T_3_ lines (*gnc*-OX7 and *gnc*-OX8) showing an obvious phenotypic change were selected for further analysis. The presence of introduced *GmGATA58* in transgenic Arabidopsis plants was first confirmed by semi-quantitative RT-PCR assay (Figure 4A). *GmGATA58* was strongly expressed in both *gnc*-OX7 and *gnc*-OX8 transgenic lines, and the endogenous *AtGNC* was expressed only in wild-type plant, but not in the *gnc* mutant and two transgenic lines (Figure 4A). Compared to the wild-type plants, the *gnc* mutant showed pale green leaves, but these pale green leaves were restored to normal green leaves of wild-type plants in both *gnc*-OX7 and *gnc*-OX8 transgenic lines irrespective of seedling or adult plants (Figure 4B and Figure S2). Moreover, the chlorophyll content in leaves also corresponded to the phenotypic complementation. The chlorophyll content in leaves was improved significantly in *gnc*-OX7 line compared to the *gnc* mutant, and even higher in *gnc*-OX7 line than wild-type plants but without significant difference (Figure 4C). These results demonstrated that *GmGATA58* could genetically complement the *gnc* mutant of Arabidopsis as *GmGATA44* did [20].

### 2.6. GmGATA58 Overexpression in Arabidopsis Increases Chlorophyll Content and Up-Regulates the Expression Levels of Chlorophyll Biosynthetic Genes

**Figure 5:**
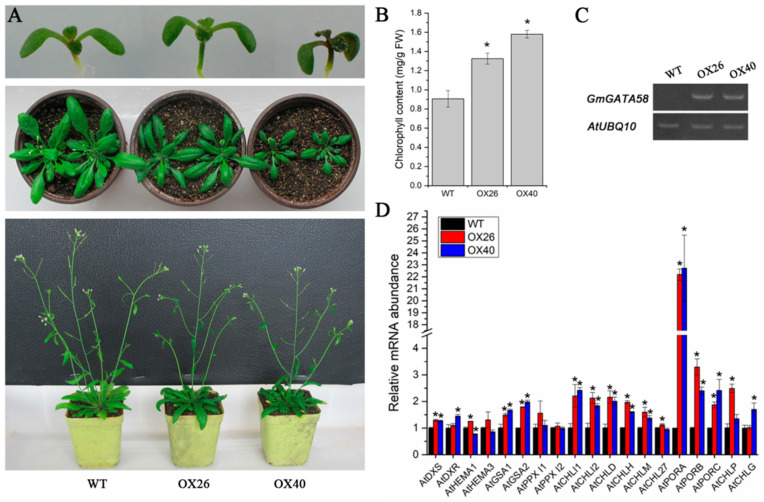
Overexpression analysis of *GmGATA58* gene in *Arabidopsis thaliana* ecotype Columbia. (**A**) Phenotypic observation of the wild-type (WT) and two independent T_3_ overexpression lines (OX26 and OX40) at one week (upper panel), four weeks (middle panel) and five weeks (bottom panel) of post-germination. (**B**) The chlorophyll content measurement in three-week-old rosette leaves of WT, the OX26 and OX40 transgenic plants. Data are presented as mean ± SD error bar (*n* = 10) from three independent measurements. Asterisks (*) indicate significant differences against the wild-type plant at *p* < 0.05. (**C**) Semi-quantitative assay of *GmGATA58* gene in rosette leaves of WT, the OX26 and OX40 transgenic plants. *AtUBQ10* gene was used as the internal reference gene. (**D**) qPCR assay of chlorophyll biosynthetic pathway genes in the leaves of WT and two overexpression lines (OX26 and OX40). Data are normalized using *AtGAPDH* as the reference gene and shown as a percentage of the expression in the wild-type plant. Asterisks (*) indicate significant differences against the wild-type plant at *p* < 0.05.

Considering the genetical complementation of *GmGATA58* in the Arabidopsis *gnc* mutant background, overexpression of *GmGATA58* in the wild-type Columbia of *A. thaliana* was also carried out. Two independent T_3_ overexpression lines (OX26 and OX40) with a high expression level of *GmGATA58* and a clear phenotypic change were selected for further analysis. The green color of cotyledons and hypocotyls of the OX26 and OX40 transgenic seedlings was clearly strengthened, compared to the wild-type control (Figure 5A, upper panel). Moreover, this deepened green color in rosette leaves maintained throughout all development stages of the OX26 and OX40 lines (Figure 5A, middle and bottom panels). Similarly, the chlorophyll content in rosette leaves was highly correlated with the green color intensity. The OX26 and OX40 transgenic lines contained a higher level of chlorophyll in leaves than the wild type (Figure 5B). In addition, the plant growth and productivity were also altered by overexpression of *GmGATA58*, which were presented in detail later. Subsequently, the expressions of introduced *GmGATA58* and chlorophyll biosynthetic pathway genes in Arabidopsis plants were also analyzed. The strong expression of *GmGATA58* in leaves of the OX26 and OX40 transgenic lines was firstly confirmed by semi-quantitative PCR assay (Figure 5C), and then the large of chlorophyll biosynthetic genes were found to be up-regulated in either or both of the OX26 and OX40 transgenic leaves by qPCR assay, including *AtGSA1* (Glutamate-1-semialdehyde 2,1-aminotransferase), *AtGSA2, AtCHLI1* (Magnesium chelatase I subunit), *AtCHLI2*, *AtCHLD* (Magnesium chelatase D subunit), *AtCHLH* (Magnesium chelatase H subunit)*, AtCHLM* (Magnesium-protoporphyrin IX methyltransferase)*, AtPORA* (Protochlorophyllide oxidoreductase), *AtPORB*, *AtPORC, AtCHLP* (Geranylgeranyl reductase) and *AtCHLG* (Chlorophyll synthase)(Figure 5D). Among them, the *AtPORA* gene which catalyzes a key step in tetrapyrrole biosynthetic pathway was most highly increased by 22-fold as well as *AtPORB* and *AtPORC* with relatively more expression levels (Figure 5D). These results indicated that *GmGATA58* plays a certain role in modulating chlorophyll biosynthesis in Arabidopsis leaves through up-regulating the expression levels of a dozen of chlorophyll biosynthetic genes.

### 2.7. Plant Growth Vigor and Productivity of Arabidopsis Are Suppressed by Overexpression of GmGATA58

**Figure 6:**
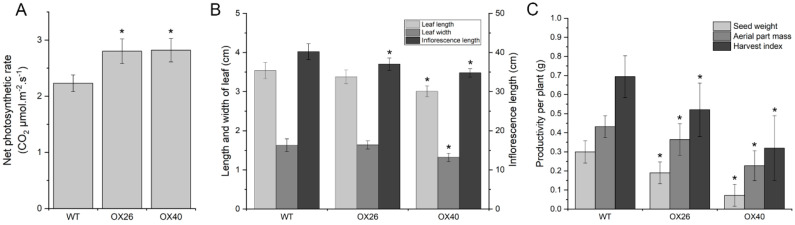
Growth and productivity alteration of the wild-type plants and two *GmGATA58* overexpression transgenic lines of *Arabidopsis thaliana*. The net photosynthetic rate (**A**), growth vigor (**B**) and productivity per plant (**C**) were measured in the wild-type (WT) plant and two overexpression lines OX26 and OX40. Data are presented as mean ± SD bar, and asterisks (*) indicate significant differences against the WT plant at the level of *p* < 0.05.

In addition to the change of chlorophyll content and leaf color mentioned above, the growth vigor and productivity of Arabidopsis plants were also altered by *GmGATA58* overexpression. Corresponding to the increase of chlorophyll content in leaves, the net photosynthetic rate was also improved slightly by ectopic expression of *GmGATA58* (Figure 6A), but the plant growth was suppressed. Compared to the wild-type plant, the two overexpression lines OX26 and OX40 revealed smaller leaves and a shorter inflorescence axis (Figure 5A and Figure 6B), and their productivities per plant including seed weight, dry mass of aerial part and their ratios (defined as harvest index) were also decreased, especially for OX40 lines (Figure 6C). Additionally, the flowering time of the overexpression lines was also significantly delayed (Figure 5A, second panel). These results suggested that ectopic expression of *GmGATA58* in Arabidopsis had a negative effect on plant growth and productivity.

### 2.8. Transcription Activation of GmGATA58 against the Promoters of Chlorophyll Biosynthetic Genes from Soybean in Transiently Transformed Arabidopsis Protoplast

**Figure 7:**
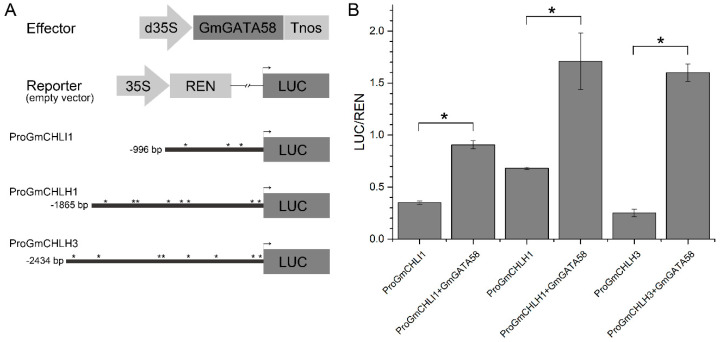
Dual-luciferase reporter assays of GmGATA58 and three promoters of chlorophyll biosynthetic genes (*GmCHLI1*, *GmCHLH1* and *GmCHLH3*) from soybean in transiently transformed protoplast of Arabidopsis. (**A**) Schematics of effector and reporter constructs for dual-luciferase reporter assay. d35S, double CaMV 35S promoter; Tnos, NOS terminator; REN, Renilla luciferase; LUC, Firefly luciferase; Asterisks (*) mean GATA binding site (A/T)GATA(A/G) in the promoter sequences, which were predicted by New PLACE tool online. Numbers indicated the length of promoter sequence upstream of the translation start codon. (**B**) Dual luciferase reporter assays of GmGATA58 against *GmCHLI1*, *GmCHLH1* and *GmCHLH3* promoters. Relative luciferase activity was determined by the ratio of LUC/REN activity. Each column represents mean ± SD error bar and asterisks (*) represent *p* < 0.05.

A dozen of chlorophyll biosynthetic pathway genes were up-regulated by ectopic expression of *GmGATA58* in Arabidopsis plants, which suggests that GmGATA58 is able to bind to their promoters and activate their transcription expression. Similarly, whether GmGATA58 has an ability to interact with and activate the promoters of key chlorophyll biosynthetic genes in soybean (*GmCHLI1*, *GmCHLH1* and *GmCHLH3*) was also investigated in transiently transformed Arabidopsis protoplast using a dual-luciferase reporter assay. The results indicated that the protoplasts co-transformed with *GmGATA58* and anyone of three soybean promoters produced a 1.5- to 5.4-fold higher value of LUC/REN fluorescence activity than the control protoplasts only transformed with the soybean promoters without *GmGATA58* TF and, among them, the transcriptional activity of *GmCHLH3* promoter was increased to the highest level (6.4-fold) by GmGATA58 activation (Figure 7). The result suggests that GmGATA58 can also bind to these three promoters of chlorophyll biosynthetic genes from soybean and activate their transcription in Arabidopsis protoplasts.

## 3. Discussion

GATA factors are considerably conserved within the GATA zinc finger domain, but not highly conserved at overall protein level. A low similarity of protein sequence of GmGATA58 with AtGNC and AtGNL was found (Figure S1), which is consistent with the sequence identity between AtGNC and AtGNL. However, GmGATA58 and GmGATA44, as well as AtGNC and AtGNL had a close phylogenetic relationship and formed a distinct cluster when the conserved GATA domains were used for phylogenetic tree construction [20]. It is well documented that both *GNC* and *GNL* play key roles in chloroplast development, modulating chlorophyll biosynthesis, chloroplast number, size and total leaf starch [12,13]. Therefore, the similar function of *GmGATA58* in transgenic Arabidopsis was also confirmed. *GmGATA58* not only played an important role in modulating chlorophyll biosynthesis, but also unexpectedly showed impacts on growth vigor, flowering time and agricultural productivity in transgenic Arabidopsis.

The reduced chlorophyll content in leaves and thus greening defect have been previously regarded as the typical phenotypes of the *gnc* and *gnl* mutants of Arabidopsis [18,28,29], while the *GNC* and *GNL* overexpressing Arabidopsis seedlings produced dark green leaves and accumulated more chlorophyll [12,13,26]. Here, our results also indicated that overexpression of *GmGATA58* in the *gnc* mutant was able to complement the greening defect (Figure 4), while overexpression in Arabidopsis wild-type plants caused an increased chlorophyll content and dark green leaves (Figure 5). The recently identified poplar *PdGNC* also revealed a similar result when overexpressed in poplar [17]. These results suggested that *GmGATA58* shared a conserved function in modulating chlorophyll biosynthesis with *GNC* and *GNL* from Arabidopsis and poplar. Correspondingly, the expression levels of the large of chlorophyll biosynthetic pathway genes in Arabidopsis overexpression lines were improved, especially *AtPORA/B/C*, *AtCHLI1/2*, *AtCHLD* and *AtCHLH* (Figure 5D). Several similar studies have also been reported previously that the expression level of certain key genes of chlorophyll biosynthesis highly correlated with the chlorophyll content of leaves in the *gnc* and/or *gnl* mutants or their overexpression transgenic plants [14,28,30]. They found that several key chlorophyll biosynthetic genes, including *AtHEMA3* (Glutamyl tRNA reductase), *AtGSA1/2*, *AtCHLI1/2*, *AtCLHM* and *AtPORBA/B/C* were regulated by *GNC* and *GNC* expressions [14,17]. However, *AtHEMA* genes, considered as a rate-limited gene for tetrapyrrole synthesis were not improved by ectopic expression of *GmGATA58* (Figure 5D). In addition, whether *GmGATA58* is able to bind to the promoters of chlorophyll pathway genes from soybean and activate their expression was also investigated in transformed Arabidopsis protoplast using a dual-luciferase reporter assay (Figure 7). All the three selected promoters from *GmCHLI1*, *GmCHLH1* and *GmCHLH3* were activated by GmGATA58. These soybean genes overlapped with the Arabidopsis chlorophyll genes which were up-regulated by ectopic expression of *GmGATA58* (Figure 5D). It is noted that soybean *PORA* gene corresponding to *AtPORA* gene with highest up-regulation was not included in dual-luciferase reporter assay, as our transcriptome results from *GmGATA44* overexpressing soybean transformants indicated that *GmPORA*, as well as *GmPORB* and *GmPORC* were not significantly changed (data not shown), but their relationships with *GmGATA58* should be further analyzed in next step.

In addition to regulating chlorophyll biosynthesis and chloroplast development, both *GNC* and *GNL* can also control a number of developmental events, including germination, expansion growth, flowering time and senescence [12,16,28,31]. Similarly, *GmGATA58* showed a wide range of functions besides regulating chlorophyll synthesis and accumulation. Overexpression of *GmGATA58* in the wild-type Arabidopsis led to delayed seed germination and flowering time as well as reduced rosette leaf size. Specifically, seeds from the transgenic lines, especially OX40 line, germinated 1–2 d slower than wild-type seeds (data not shown), and flowering time of OX26 and OX40 lines was obviously delayed compared to that of the wild type. These phenotypic changes are consistent with the previous reports that seeds germinated faster, rosette leaf size enlarged and plants flowered earlier for the *gnc* and *gnl* mutants [28], whereas the *GNC* and *GNL* overexpressing plants showed a slower seed germination and flowering time, compared to the wild-type plants [26,32]. In addition, overexpression of other LLM domain-containing B-GATA factors, including *SlGATA4*, *SlGATA5* and *SlGATA7* from tomato (*Solanum lycopersicon*) as well as *BdGATA4* and *BdGATA6* from Brachypodium (*Brachypodium distachyon*) in Arabidopsis, also produced a similar phenotype [26]. They hypothesized that the LLM domain-containing B-class GATA TFs were functionally conserved among different species. Our results presented here also strongly supported this hypothesis.

It is interesting and unexpected that the net photosynthetic rate in leaves was slightly improved, but the plant growth and agricultural productivity per plant were clearly reduced in the *GmGATA58* overexpression lines (Figure 5 and Figure 6), which was strongly contrary to the report of *PdGNC* [17]. Overexpression of *PdGNC* in poplar led to faster growth, higher biomass accumulation and an increase in chlorophyll content, photosynthetic rate and plant height. In this study, the transgenic plants overexpressing *GmGATA58* grew weakly at both young and adult stages with smaller leaves and shorter inflorescences, compared to the wild-type control, which possibly caused a low seed yield and biomass of aerial part per plant (Figure 6). In fact, a lot of the evidence showed that there is no correlation between the yield of a wide range of crops and photosynthesis, and the yield is limited by sinks for photosynthates, but not by photosynthetic capacity [4]. The improvement of photosynthetic efficiency also contributes to a greater yield. Although the net photosynthetic rate is slightly improved in leaves by overexpression of *GmGATA5*8, photosynthetic energy conversion efficiency and photosynthate transportation efficiency may not be up-regulated. Moreover, it is suggested that *GATA* genes share both conserved and diverged functions between herbaceous and woody plants as *PdGNC* showed a similar and a different roles with *AtGNC* and *AtGNL* [17]. Perhaps, that is why *GmGATA58*-expressing transformants revealed some contrary phenotypic traits with the *PdGNC*-expressing transformants.

In plants, photosynthesis takes place primarily in chloroplasts of leaves, which contain chlorophyll. Since *GmGATA58* played an important role in modulating chlorophyll biosynthesis, it should be highly expressed in leaf tissue. Our qPCR assay and GUS staining assay results also supported this assumption (Figure 2A and Figure 3). As previously demonstrated, *GNC* and *GNL* paralogs are induced by nitrogen sources, cytokinin and light, but suppressed by GA and darkness [13,18,28,33]. However, the expression pattern of *GmGATA58* in response to these factors showed partial difference, although the similar light- and phytohormone-responsive elements were found in the *GmGATA58* promoter sequence (Table S1). The transcript level of *GmGATA58* was also induced by high nitrogen condition and suppressed by low nitrogen status (Figure 2B). Nitrogen conditions have a marked effect on chlorophyll synthesis and they are highly correlated with each other [34]. *GmGATA58* was remarkably down-regulated by 2, 4-D (Figure 2D), which agreed with the report on *GNC* and *GNL* [18,35]. By contrast, *GmGATA58* expression was suppressed by light and 6-BA (Figure 2C,D) and promoted by GA_3_ treatment (Figure 2D), which were partially contrary to the previous report for *GNC* and *GNL*. Light is an important external signal and plant hormones as internal signals coordinate control plant growth and development, including chlorophyll biosynthesis [36]. The de-etiolation process takes place when dark-grown seedlings are exposed to light, accompanied with the accumulation of chlorophyll, and multiple endogenous phytohormones are also involved. Previous studies showed that *AtGNC/GNL* positively responded to light and cytokinin, and negatively to auxin and gibberellins [28,33,35]. In this study, the reasonable explanation for the suppression of *GmGATA58* by light and the promotion by GA_3_ is still unknown, and there may be other regulatory proteins affecting *GmGATA58* expression via interaction or coordination. It is also possible that *GmGATA58* is functionally redundant in regulating chlorophyll biosynthesis as its paralogous GmGATA44 also played an important role in chlorophyll biosynthesis [20]. In summary, *GmGATA58* was induced by nitrogen and responded to light and hormone treatments, but the responses did not exactly coincide with Arabidopsis *GNC* and *GNL*.

## 4. Materials and Methods

### 4.1. Plant Materials

Plants of soybean (*Glycine max*) cultivar ‘TianLong No.1′ were grown under white light (200 μmol·m^−2^·s^−1^) and about 60% relative humidity with a 16 h light/8 h dark cycle at 28 °C in a plant growth chamber and various tissues including root, stem, leaf, petiole, flower, young pod, immature seed, pod wall of immature seed and mature seed were collected for qPCR assay. Soybean seedling in response to nitrogen treatment was carried out following the method by Zhang et al. [20] with minor modification. In brief, the 5-day-old soybean seedlings were planted in the mixture of vermiculite:perlite = 2:1 for 4 d and then the cotyledons of seedlings were cut off prior to nitrogen treatment. The seedlings were irrigated with half Hoagland solution with 7.5 mM nitrogen as normal nitrogen condition for 4 d, and then transferred to 10 times-diluted half Hoagland solution with 0.75 mM nitrogen as low nitrogen stress (N−) or continued to be kept in the normal nitrogen condition (N+). The nutritional solution was refreshed every 4 days and the first fully expanded leaves were harvested for qPCR assay at different time points after treatment. For light treatment, the 15-day-old soybean seedlings were grown in the continuous dark for 3 d and then transferred to continuous light condition (about 100 μmol·m^−2^·s^−1^) for up to 3 d. The first fully expanded leaves were harvested for qPCR assay. As for phytohormones treatments, the 15-day-old soybean seedlings grown in compost plastic pots in a naturally illuminated glasshouse were sprayed with 100 μM 6-BA, 100 μM 2, 4-D or 100 μM GA_3_ hormones. The seedlings sprayed with distilled water were used as control. The first fully expanded leaves from the apical bud of soybean were collected at 0, 2, 4, 8, 12 and 24 h after treatment.

The wild-type Columbia and *gnc* mutant of *A. thaliana* were used for plant transformation. They were grown under white light (100 μmol·m^−2^·s^−1^) and 60% relative humidity with a 16 h light/8 h dark cycle at 24 °C. The *gnc* mutant contains a T-DNA insertion in the second exon of *AtGATA21* (*At5g56860*) and produces greening defect with reduced chlorophyll content in pale green leaves. The *gnc* mutant line was obtained from the Arabidopsis Biological Resource Center (Stock No: SALK_001778). Arabidopsis plants were grown in a plant growth chamber at 24 °C under the condition of 16 h of light and 8 h of dark.

### 4.2. DNA and RNA Extraction

Genomic DNA from young leaves of soybean and *A. thaliana* was isolated using CTAB method [37]. Total RNA was extracted from various tissues of soybean and young leaves of transgenic Arabidopsis plants using Trizol reagent (Invitrogen, Carlsbad, CA, USA) according to the manufacturer’s instruction. The quality and quantity of nucleic acids were evaluated by agarose gel electrophoresis and Epoch microplate spectrophotometer (BioTek, Winooski, VT, USA).

### 4.3. Isolation of GmGATA58 gene

Based on the sequence of *Glyma.17G055200* (Phytozome, v12.0), the full-length cDNA clone of *GmGATA58* was isolated from leaf cDNA template of soybean variety ‘TianLong No.1′ using the gene-specific primers (Table S2). The PCR product was cloned into the pMD18-T vector (Takara, Dalian, China and sequenced for further confirmation).

### 4.4. Subcellular Localization of GmGATA58

The coding region of *GmGATA58* was amplified from pMD18-GmGATA58 recombinant plasmid using the gene-specific primers (Table S2) and then subcloned into the pEGAD binary vector with *Sma* I and *Bam*H I double digestion to generate the CaMV 35S-GFP::GmGATA58 construct for subcellular localization assay. In this construct, GmGATA58 protein was fused to the C-terminus of GFP protein under the control of the CaMV 35S promoter. This construct was introduced into *Agrobacterium tumefaciens* strain EHA105 by the freeze–thaw method. Agro-infiltration transient expression in leaves of *Nicotiana benthamiana* was used for subcellular localization assay of GmGATA58. The plasmid construct coding AtFib2-mCherry fusion protein was used as a nucleolar marker [27]. Leaves of *N. benthamiana* were infiltrated with *Agrobacterium* cultures to transiently express GFP protein alone or GFP::GmGATA58 fusion protein in combination with the AtFib2-mCherry nucleolar marker. After 3 d of infiltration, GFP (green) and mCherry (red) fluorescence signals were observed using a confocal laser scanning microscopy (Leica, Wetzlar, Germany).

### 4.5. Isolation and Analysis of GmGATA58 Promoter

A promoter region upstream of the start codon ATG (2013 bp) of *GmGATA58* was amplified from soybean genomic DNA using the promoter-specific primers (Table S2) and subcloned into the pCXGUS-P vector [38] to generate the pCXGUS-GmGATA58p construct, in which the reporter *GUS* gene was expressed under the control of the *GmGATA58* promoter. This construct was then introduced into *Agrobacterium tumefaciens* strain GV3101 by freeze–thaw method and transformed into *A. thaliana* Columbia wild-type plants using floral dip method [39]. For GUS histochemical staining, various tissues from wild-type and transgenic plants carrying the *GmGATA58* promoter were harvested, treated with 90% acetone for 20 min, rinsed with distilled water and incubated with GUS staining solution at 37 °C overnight in dark. Finally, these tissues were destained using 70% ethanol prior to photographing under a microscope. In addition, the putative *cis*-acting regulatory elements in the promoter sequence of *GmGATA58* were predicted using PlantCARE database [40].

### 4.6. Overexpression Vector Construction and Arabidopsis Transformation

The complete coding region of *GmGATA58* was amplified from pMD18-GmGATA58 recombinant plasmid using the gene-specific primers (Table S2) and inserted into the intermediate vector pGWC, which can be digested with *Ahd* I enzyme to generate a T-vector. Subsequently, *GmGATA58* was transferred from pGWC vector to pB2GW7 vector by LR reaction (Invitrogen, Carlsbad, CA, USA) to generate a pB2GW7-GmGATA58 overexpression construct, in which *GmGATA58* expression was driven by the CaMV 35S promoter. This overexpression construct was introduced into *Agrobacterium tumefaciens* strain GV3101 by freeze-thaw method. The wild-type Columbia plants and the homozygous mutant *gnc* of *A. thaliana* were used for genetic transformation via floral dip method [39].

Basta-resistant plants were further confirmed by PCR using the CaMV 35S promoter specific primer with the *GmGATA58* reverse primer. The Basta resistant screening was used in genetic segregation analysis to select homozygous lines. The expression level of *GmGATA58* in transgenic lines was analyzed by semi-quantitative RT-PCR assay. Finally, a total of four independent homozygous T_3_ lines with a high expression level of *GmGATA58* were selected for further study, including two *GmGATA58* genetical complement lines in the *gnc* mutant background (*gnc*-OX7 and *gnc*-OX8) and two *GmGATA58* overexpression lines in wild-type background (OX26 and OX40).

### 4.7. Semi-Quantitative RT-PCR and Quantitative PCR Analysis

After treated with RNase-free DNase I (Thermo Fisher Scientific, Waltham, MA, USA), one microgram of total RNA was used to synthesize fist-strand cDNA using M-MLV reverse transcriptase (Promega, Madison, WI, USA) according to the supplier’s manual. The expression levels of *AtGNC* and *GmGATA58* in leaves of Arabidopsis lines were analyzed by semi-quantitative RT-PCR. PCR amplification was performed using the following program: one denaturation cycle at 94 °C for 5 min, 31 cycles of 94 °C for 30 s, 56 °C for 30 s, and 72 °C for 30 s, and a final extension at 72 °C for 10 min. The amplified products were analyzed by electrophoresis on 1.0% agarose gel.

A qPCR assay was used to analyze the expression pattern of *GmGATA58* in various tissues of soybean and soybean leaves under nitrogen, phytohormones and light treatments, and also the expression levels of chlorophyll biosynthetic genes in transgenic overexpression lines of Arabidopsis. The qPCR assay was carried out using SYBR Premix Ex Taq II kit (Takara, Dalian, China, following the manual’s recommendation, and run on ABI7500 Fast Real-Time PCR equipment (Applied Biosystems, Foster, CA, USA). In brief, the reaction mixture contained 10 μL of 2× SYBR Premix Ex Taq mix, 0.3 μM each of forward and reverse primers, and 2 μL of 5-fold-diluted first-strand cDNA template. The qPCR program was as follows: 95 °C for 30 s, 40 cycles of 95 °C for 3 s and 60 °C for 30 s, and a default melting curve program. Three biological replicates were performed for each sample. Soybean *ACT11* (*Glyma.18G290800*) gene and Arabidopsis *GAPDH* (*At3g26650*) gene were used as the internal controls for qPCR assay in soybean and Arabidopsis, respectively. The relative expression level of gene was analyzed using 2^−ddCt^ method [41]. All primers were designed using Primer 5.0 software and listed in the Supplementary Table S2.

### 4.8. Determination of Chlorophyll Content, Photosynthetic Rate and Growth in Arabidopsis

Determination of chlorophyll content and photosynthetic rate in rosette leaves of Arabidopsis was carried out after three weeks of seed germination. The chlorophyll content was measured using a spectrophotometric method as previously described [42]. The net photosynthetic rate was measured using a LI-6400-XT portable photosynthesis system with a plant Arabidopsis chamber (Li-Cor, Lincoln, NE, USA) following the method by Xin et al. [43]. For measurement of chlorophyll content and net photosynthetic rate, a total of ten independent samples for each line were measured, and each sample was repeated in triplicate. In addition, the productivity per plant for each line, including seed weight, dried weight of aerial part of plant and their ratios (referred to harvest index) was determined from 25 to 30 independent individual plants.

### 4.9. Dual-Luciferase Assay in Transiently Transformed Protoplast of Arabidopsis

The promoter regions of *GmCHLH1* (*Glyma.03G137000*) in length of 1865 bp, *GmCHLH3* (*Glyma.19G139300*) in length of 2434 bp and *GmCHLI1* (*Glyma.13G232500*) in length of 996 bp upstream of the start codon ATG were amplified from soybean genomic DNA using the promoter-specific primers (Table S2) and cloned into pGreen II 0800-LUC vector to generate *ProGmCHLH1::LUC*, *ProGmCHLH3::LUC* and *ProGmCHLI1::LUC* reporter plasmids. pGreenⅡ0800-LUC vector without promoter insertion was used as a control. The effector plasmid was constructed by cloning the full-length coding region of *GmGATA58* into the pAN580 vector under the control of the double 35S promoter. As described previously [44], the reporter plasmid was co-transformed with the effector plasmid or transformed alone into Arabidopsis protoplasts. The *Pro35S:REN* gene (*Renilla luciferase*) in the pGreenⅡ-LUC vector was used as an internal control. The Dual-Luciferase Reporter assay system (Promega, Madison, WI, USA) was used to determine the ratio of firefly luciferase to Renilla luciferase (LUC/REN) to indicate the promoter activities.

### 4.10. Statistical Analysis

Data were presented as mean ± SD (standard deviation) bar. Statistical separation of treatment means was by analysis of variance (ANOVA) using Originpro 2020b (OriginLab Corporation, Northampton, MA, USA) at the level of *p* < 0.05. Graphs were also created using Originpro 2020b (OriginLab Corporation, Northampton, MA, USA).

## 5. Conclusions

As an LLM domain-containing B-class GATA TF, *GmGATA58* was demonstrated to be involved in regulating chlorophyll biosynthesis in Arabidopsis as overexpression of *GmGATA58* not only genetically complemented the greening defect of the *gnc* mutant, but also improved chlorophyll content in transgenic leaves through up-regulation of the expression levels of most of chlorophyll biosynthetic genes. Meanwhile, the net photosynthetic rate in transgenic leaves was also slightly improved, but unexpectedly the Arabidopsis growth and productivity were clearly suppressed. In addition, *GmGATA58* was predominantly expressed in leaves and responded to nitrogen, light and phytohormone treatments.

## Supplementary Materials

The following are available online at https://www.mdpi.com/2223-7747/9/8/1036/s1. Figure S1: Multiple alignment of deduced amino acids of GmGATA58 with other three GATA factors. Figure S2: Restoration of greening defect of *Arabidopsis thaliana gnc* mutant by *GmGATA58* overexpression. Table S1: Putative *cis*-acting regulatory elements predicted in the *GmGATA58* promoter sequence. Table S2: Primers used in this study for cloning, vector construction, semi-quantitative RT-PCR and qPCR assay.
