# Supplementary material for: A GATA Transcription Factor from Soybean (Glycine max) Regulates Chlorophyll Biosynthesis and Suppresses Growth in the Transgenic Arabidopsis thaliana
Source: Plants (Basel). 2020 Aug 15;9(8):1036. doi: 10.3390/plants9081036 (PMC7464611; doi:10.3390/plants9081036)
Supplement: Supplementary file 1 [file plants-09-01036-s001.pdf]

plants

MDPI

Article

A GATA Transcription Factor from Soybean (*Glycine max*) Regulates Chlorophyll Biosynthesis and Suppresses Growth in the Transgenic *Arabidopsis thaliana*

Chanjuan Zhang [1], Yi Huang [1], Zhiyuan Xiao [1], Hongli Yang [1], Qingnan Hao [1], Songli Yuan [1], Haifeng Chen [1], Limiao Chen [1], Shuilian Chen [1], Xinan Zhou [1,*] and Wenjun Huang[2,3,*]

Supplementary Materials:

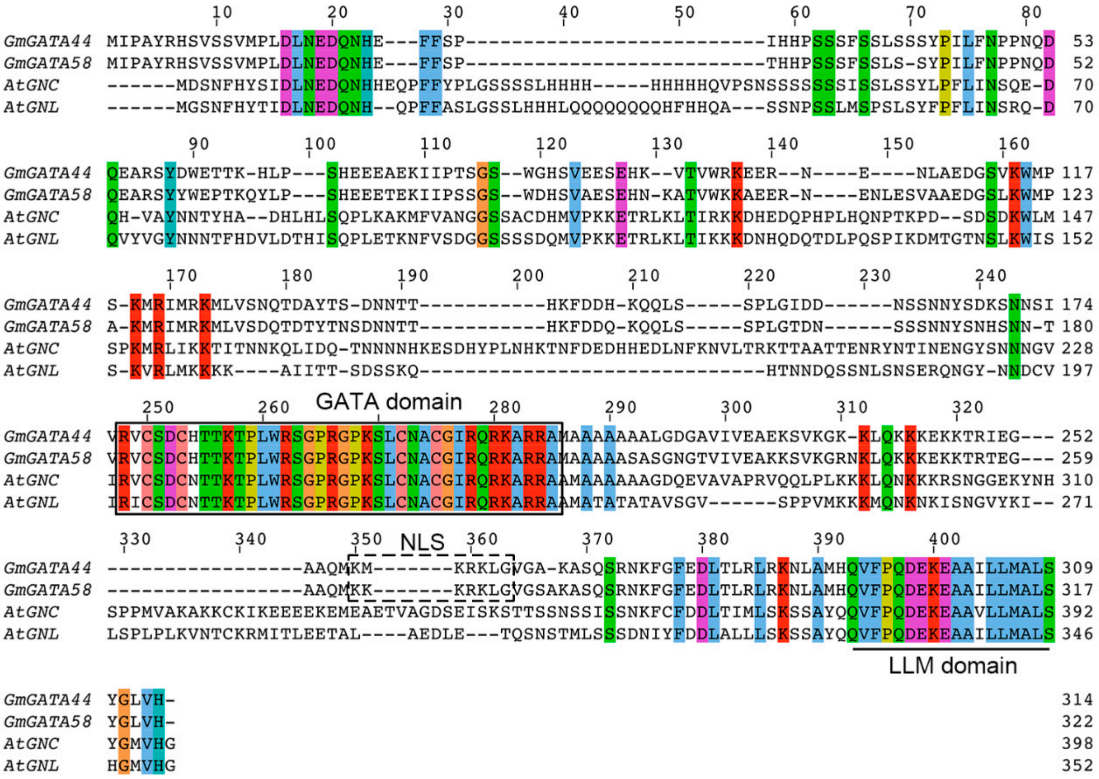

Figure S1. Multiple alignment of deduced amino acids of GmGATA58 with other three GATA factors. The highly conserved GATA domain, conserved leucine-leucine-methionine (LLM) domain and nuclear localization signal (NLS) were indicated. The protein alignment was generated based on the full-length protein sequences using the Clustal Omega algorithm at the EMBL-EBI website (http://www.ebi.ac.uk/Tools/msa/clustalo/) with the default settings. Protein sequences were retrieved from GenBank and their accession numbers were as follows: *Glycine max* GATA58 (XP_003550634.1) and GATA44 (XP_003543725.1), *Arabidopsis thaliana* GNC (NP_200497.1) and GNL (NP_194345.1).

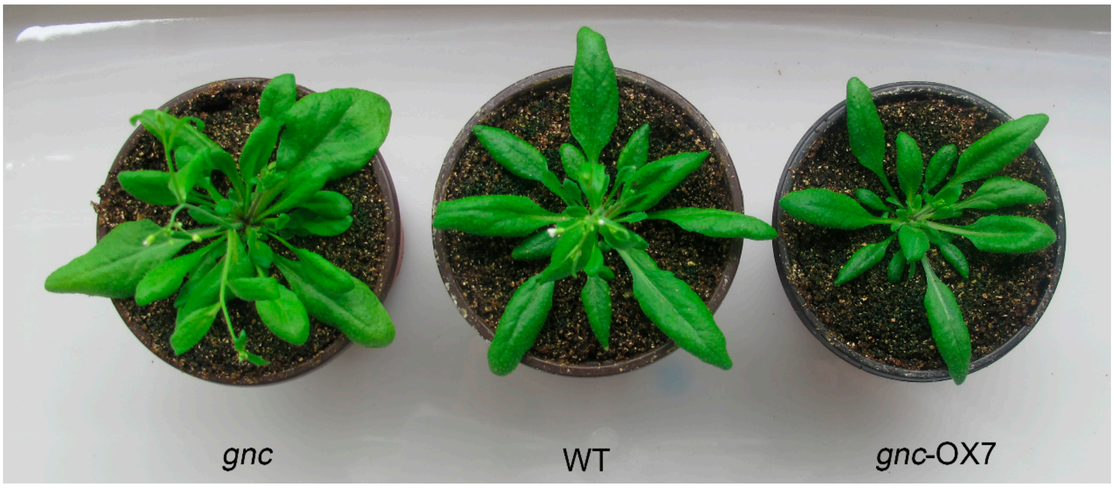

Figure S2. Restoration of greening defect of *Arabidopsis thaliana gnc* mutant by *GmGATA58* overexpression. Phenotypic comparison of wild-type (WT), *gnc* mutant and overexpression of *GmGATA58* in the *gnc* mutant background (gnc-OX7).

Table S1. Putative *cis*-acting regulatory elements predicted in the *GmGATA58* promoter sequence.

No.	*Cis*-Element Name	Sequence	Function	Element Number
**1**	ABRE (ACGT-containing ABA Response Element)	CACGTG	abscisic acid responsiveness	1
**2**	ACE (ACGT-Containing Element)	AAAACGTTTA	light responsiveness	1
**3**	AE-box (Activating Element)	AGAAACTT	light response	1
**4**	ARE (Anaerobic Response Element)	TGGTTT	anaerobic induction	2
**5**	AT1-motif	ATTAATTTTACA	light responsive module	1
**6**	Box 4	ATTAAT	light responsiveness	3
**7**	CATT-motif	GCATTC	light responsive element	2
**8**	chs-CMA1a (Chs Conserved DNA Module Array 2)	TTACTTAA	light responsive element	1
**9**	Circadian (Circadian Control)	CAAAGATATC	circadian control	1
**10**	G-Box	CACGTG	light responsiveness	1
**11**	G-box	GACATGTGGT	light responsiveness	1
**12**	GA-motif	AAGGAAGA	light responsive element	1
**13**	GATA-motif	AAGATAAGATT	light responsive element	1
**14**	GCN4_motif	TGAGTCA	endosperm expression	1
**15**	HSE (Heat Shock Element)	AAAAAATTTC	heat stress responsiveness	2
**16**	I-box	AGATAAGG	light responsive element	1
**17**	MBS (MYB Binding Site)	T(C)AACTG	drought-inducibility	4
**18**	MRE (MYB Recognition Element)	AACCTAA	light responsiveness	2
**19**	O2-site (Opaque-2 site)	GT(A)TGACG(A)TGA	zein metabolism regulation	2
**20**	P-box (Pyrimidine Box)	CCTTTTG	gibberellin-responsive element	1
**21**	Skn-1_motif	GTCAT	endosperm expression	5
**22**	Sp1	CC(G/A)CCC	light responsive element	1
**23**	TATC-box	TATCCCA	gibberellin-responsiveness	1
**24**	TCA-element	CCATCTTTTT	salicylic acid responsiveness	1
**25**	TCCC-motif	TCTCCCT	light responsive element	2
**26**	TCT-motif	TCTTAC	light responsive element	1
**27**	TGA-element	AACGAC	auxin-responsive element	1

Table 2. Primers used in this study for cloning, vector construction, semi-quantitative RT-PCR and qPCR assay.

Gene Name	Accession No.	Primer Sequence (Forward/Reverse)	Note
GmGATA58	Glyma.17G055200	GTGTTTTTATATGTGTGTTCCTCCA; AGTGAAGTGGTGGTAGCTCTAGC	Full-length cDNA cloning
		TCCCCCGGGATGATTCCAGCCTATCGCCA; CGGGATCCTCAATGAACAAGGCCATAAGATAA	Subcellular localization assay
		TGATGTTAGGTTAGCATTTACATGTG; GATGGAGGAACACACATATAAAAAC	Promoter isolation
		ATGATTCCAGCCTATCGCC; TCAATGAACAAGGCCATAAGATA	Coding region amplification for plant transformation
		CGGAAGATGTTGGTGTCGG; TTGAATAGTTGTTGCTGCTGCTG	qPCR assay
		GGAGCTAGCTCTAGAATGATTCCAGCCTATCGCC; ATGTTTGAACTGCAGTCAATGAACAAGGCCATAAGATA	Dual-luciferase reporter assay
		GAATGAAAATCTTGAATCAGTTGC; ACAATTACTGTTCCATTTCCCG	Semi-quantitative RT-PCR assay
AtGNC	At5g56860	GCGTGATTAGGGTTTGTTCG; GCCACCATTGGAGGAGAGT	Semi-quantitative RT-PCR assay
AtUBQ10	At4g05320	GGCTGATTACAATATCCAGAAGG; ATCCTCCAACTGCTTTCCG	Semi-quantitative RT-PCR assay
GmCHLH1	Glyma.03G137000	ACGGTATCGATAAGCTTTGATCGCCCTCACCTCGT AGAACTAGTGGATCCTGATAATAATAATCAAGCTAAGTAGGAAAC	promoter isolation for dual-luciferase reporter assay
GmCHLH3	Glyma.19G139300	ACGGTATCGATAAGCTTTTAAGCTACTTCAGTCGATCCTTGA; AGAACTAGTGGATCCTGATAAATAATCAAGCTAAGTAGGAAACA	promoter isolation for dual-luciferase reporter assay
GmCHLI1	Glyma.13G232500	ACGGTATCGATAAGCTTTCATAATCAATGGCACCCACTAAC; AGAACTAGTGGATCCAACCGGAGAAGAGAAGAGAAGACA	promoter isolation for dual-luciferase reporter assay
GmACT11	Glyma.18G290800	ATCTTGACTGAGCGTGGTTATTCC; GCTGGTCCTGGCTGTCTCC	qPCR assay
AtDXS	At4g15560	AACTTACTTTGCGGAGGCATTAG; CATCTTGTTGGGAAGCGACG	qPCR assay
AtDXR	At5g62790	GCCTGATATGCGTTTACCGATTC; AGAACTCCAGTCATTGTGCCTCC	
AtHEMA1	At1g58290	CACGGGTTTACAATGTGGACG; CCCTCCATGCTTCAAACTGTG	
AtHEMA3	At2g31250	GACAAAAAGACGAGGGAAGCAG;	

		GCTGCTACCAGTCCCATCGTA
AtGSA1	At5g63570	ATACTTTGGAATCACGCCTGATC;<br>ATCTCCATAATATCACGTCTACCACC
AtGSA2	At3g48730	GACCGATGTATCAAGCTGGTACG;<br>AAGTATTCATATGTCCCTGGCTGAC
AtPPX I1	At4g01690	TGAGCATGAAAGCAGCGTTTG;<br>AAGTCCCTTCCTGAAAGAACCAA
AtPPX I2	At5g14220	CAACTTTTATTGGTGGGAGTAGGAA;<br>TGGTTGACAGACACGGGTTCA
AtCHLI1	At4g18480	AGACATAGTGACTAACAGAGCAGCAA;<br>AGCAGTTAGGGATAACGGTTGC
AtCHLI2	At5g45930	GACGCCGAGCTGAGAGTTAAGA;<br>CTTCTTGCAGTTGTAATCTGCTCC
AtCHLD	At1g08520	CCGATGGTCGAGCCAACATTA;<br>TTGGACGTGGGTCTAGGAGCA
AtCHLH	At5g13630	ACACCAATCCCAACTCCTTCAG;<br>TCAGCGGAAGTGTCCCAGTAG
AtCHLM	At4g25080	TGAGATGAAGGCAAAGGCACA;<br>GTCTGCTTTGTTCTGCGGGTA
AtCHL27	At3g56940	GATTTCGCCGAGTTTGAGCC;<br>AAATCAAGAACCAGCCGTAAACTC
AtPORA	At5g54190	TGTATTGGAGCTGGAACAAGACC;<br>CCAAAGGTTGAAACACCGAGG
AtPORB	At4g27440	AAGGCTCGTAAAGTGTGGGAGA;<br>TCGATTGGTACCGAGAGGTGTC
AtPORC	At1g03630	ACACATACCGCTGTTTCGGC;<br>CAATACACTCCTGACTTCCCAAGA
AtCHLP	At1g74470	GATGCTGGTGATTACGACTACGC;<br>CACTTAGGGAACACCCAACCATAG
AtCHLG	At3g51820	CTCTTACGCCAGATGTTGTTGTTC;<br>TGCCAAAAGCTACTGGGAGAGA
AtGAPDH	At3g26650	CTTGGAAGGAGCTAGGAATTGACA;<br>ATGTGTTTCCCTGCACCTTCTC

© 2020 by the authors. Licensee MDPI, Basel, Switzerland. This article is an open access article distributed under the terms and conditions of the Creative Commons Attribution (CC BY) license (http://creativecommons.org/licenses/by/4.0/).
